# Diagnostic Genetics at a Distance: Von Hippel-Lindau Disease and a Novel Mutation

**DOI:** 10.1155/2013/189196

**Published:** 2013-08-26

**Authors:** Clare Brookes, Debra O. Prosser, Jennifer M. Love, R. J. McKinlay Gardner, Donald R. Love

**Affiliations:** ^1^Diagnostic Genetics, LabPLUS, Auckland City Hospital, P.O. Box 110031, Auckland 1148, New Zealand; ^2^Genetic Health Service New Zealand-Northern Hub, Auckland City Hospital, Private Bag 92024, Auckland 1142, New Zealand; ^3^Clinical Genetics Group, Department of Paediatrics and Child Health, University of Otago, P.O. Box 56, Dunedin 9054, New Zealand; ^4^School of Biological Sciences, University of Auckland, Private Bag 92019, Auckland 1142, New Zealand

## Abstract

Genetic testing at a distance is commonplace where members of a family with a segregating germline mutation are geographically separated. For the most part, this challenge is addressed through the intervention of health professionals in taking and/or processing blood samples for subsequent couriering of DNA to a referral laboratory. In some circumstances, however, the collecting of pivotal clinical material may involve direct patient involvement. We describe such a situation where noninvasive saliva samples were provided by members of a family manifesting Von Hippel-Lindau (VHL) disease. The analysis identified a novel mutation in the *VHL* gene that was used to exclude other family members as being at risk of VHL disease.

## 1. Introduction

Von Hippel-Lindau (VHL) disease is an autosomal dominant familial cancer syndrome with a prevalence of approximately 1/39,000 births [1]. The most common tumours in VHL disease are renal cell carcinomas (RCCs), retinal and central nervous system haemangioblastomas, phaeochromocytomas, pancreatic islet tumours, and endolymphatic sac tumours (ELSTs) [2].

In patients with a family history of VHL, a diagnosis can be made with the finding of a single retinal or cerebellar haemangioblastoma, a phaeochromocytoma, or an RCC [3]. In the absence of a family history, a diagnosis can be made with two or more retinal or cerebellar haemangioblastomas or one haemangioblastoma and one visceral tumour [3]. Families can be characterised by the absence (type 1) or presence (type 2) of phaeochromocytomas [4].

VHL disease is due to germline mutation in the *VHL* tumour suppressor gene, which is located on the short arm of chromosome 3 [5]. The gene comprises three exons and encodes for two proteins: 30 kD (pVHL30, NP_000542.1 expressed from NM_000551.3; transcript variant 1) and 18 kD (pVHL18, NP_937799.1 expressed from NM_198156.2; transcript variant 2) [6]. The shorter pVHL18 protein is generated from alternative translation that starts from an internal methionine at codon 54 [6]. In nude mouse xenograft assays, both the pVHL30 (wildtype) and pVHL18 proteins function as tumour suppressors when introduced into clear-cell RCC cell lines lacking functioning *VHL* genes [6].

Over 1000 somatic and germline *VHL* gene mutations have been documented [7], although the web-accessible database at http://databases.lovd.nl/genomed/home.php?select_db=VHL, updated January 14, 2013, shows 430 unique variants with 18 of these identified using tumour samples. Nordstrom-O'Brien et al. report a mutation spectrum of 43.2% in exon 1, 17% in exon 2, and 39.8% in exon 3 [7]. Missense mutations are the most common type of mutation (61%), followed by frameshift (15.7%), nonsense (13.2%), in-frame insertions/deletions (6.6%), and splicing mutations (3.5%) (large deletions and rearrangements are not included). An analysis of 114 VHL families (77% type 1 and 23% type 2) found that 97% of small deletions/insertions, nonsense, or deletion mutations were identified in type 1 families, and that mutations of these types accounted for 56% of all type 1 mutations [8]. 96% of the mutations identified in the type 2 families were missense mutations, with 10/23 mutations occurring at codon 238. Truncating mutations have also been shown to lead to a higher risk of VHL patients developing RCC [9].

The detection of a mutation in a proband allows the identification of mutation carriers among family members who have not yet displayed clinical symptoms. Regular tumour surveillance for mutation-positive family members can then be offered.

We present here a case in which *VHL* gene predictive testing was requested by a New Zealand family who had a reported family history of VHL disease. Genetic testing for this family had not previously been performed, and the only immediate family member who was alive and symptomatic was in Iraq. We describe the ability to test international saliva samples that were taken in the absence of health professionals and shipped with no active intervention regarding temperature, and the results of the molecular analysis that revealed a novel mutation in the *VHL* gene.

## 2. Methods

### 2.1. DNA Extraction

Saliva samples from two family members were collected in Iraq using Oragene•ONE (ON-500) (DNA Genotek) collection kits, which were posted by ordinary airmail to New Zealand, without temperature protection. Genomic DNA (gDNA) was isolated using the method described by the manufacturer (DNA Genotek, Inc., Ontario, Canada).

Peripheral blood in EDTA was collected from two family members in New Zealand. gDNA was isolated using the Gentra Puregene DNA Extraction kit (Qiagen Pty Ltd). Spectrophotometric analysis of gDNA samples was undertaken using a Nanodrop ND-1000 spectrophotometer (Nanodrop Technologies, Wilmington, DE, USA), and gDNAs were electrophoretically separated in a 2% E-gel (Life Technologies Corporation) for 7 minutes.

### 2.2. Primer Design

The mRNA sequence of the *VHL* gene was identified using the UCSC genome browser (http://genome.ucsc.edu/). This website provides a direct link to ExonPrimer for the design of primers flanking coding exons. ExonPrimer is a Perl script that uses a combination of Primer3 and Blat to design intronic primers against a cDNA of interest. All primers were checked for single nucleotide polymorphisms using the software tool available from the National Genetic Reference Laboratory, Manchester (https://ngrl.manchester.ac.uk/SNPCheckV3/snpcheck.htm). The primers were tailed with M13 sequences and were synthesised by Invitrogen Ltd (Figure 5).

### 2.3. PCR

PCR was performed using 1U Faststart Taq DNA polymerase (Invitrogen Ltd), 50 ng genomic DNA, 2 mM MgCl_2_, and 0.8 *μ*M forward and reverse primers, with the following cycle conditions: 95°C for 4 min, 35 cycles of 94°C for 45 s, 60°C for 30 s, 72°C for 30 s, and a final extension at 72°C for 10 min. All amplicons amplified efficiently under these conditions.

### 2.4. Sequencing

5 *μ*L of each PCR was cleaned with ExoSAP-IT (Affymetrix) prior to bidirectional DNA sequencing using M13 forward and reverse primers and Big-Dye Terminator v3.0 (Applied Biosystems Ltd). 20 *μ*L of sequenced product was purified using Clean-Seq (Agencourt). 15 *μ*L of purified product was then subjected to capillary electrophoresis using an Applied Biosystems model 3130xls genetic analyser.

### 2.5. Sequence Analysis

The analysis of sequence traces was performed using Variant Reporter v1.0 (Applied Biosystems). Genbank NM_000551.3 was used as the reference sequence, with cDNA number +1 corresponding to the A of the translation initiation codon. Amino acid numbering begins at the first amino acid in RefSeq accession number NP_000542.1. Variant Reporter uses advanced algorithms and quality metrics to automate the detection of variants and to streamline the analysis process. 

## 3. Results

### 3.1. Family History

**Figure 1:**
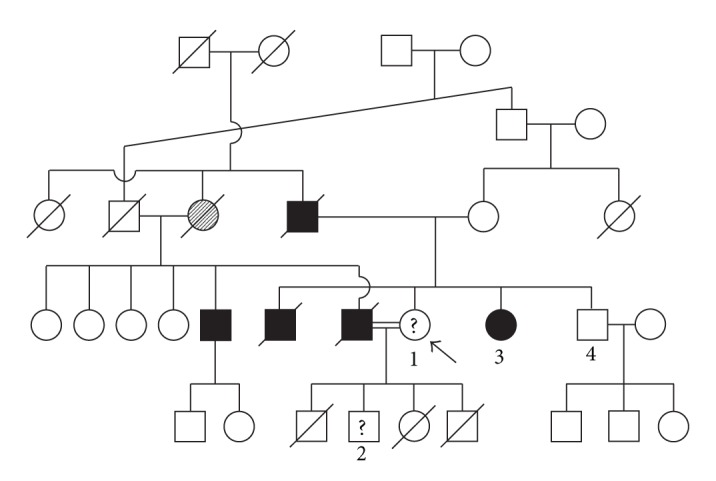
Family pedigree. Patients 1 and 2 = New Zealand; patients 3 and 4 = Iraq. The three deaths in the siblings of patient 2 were due to a coincidental autosomal recessive disease (Hurler syndrome). Filled symbol = presumed VHL; shaded symbol = presumed heterozygote (no known overt disease); ? = apparently unaffected, seeking genetic advice.

A mother and son, neither with any symptoms of VHL, presented to Genetic Health Service New Zealand with a family history of VHL and consanguinity (they are identified as patients 1 and 2 in Figure 1). The anecdotal information provided to us was as follows. Patient 3 had surgery for a brain tumour at approximately 30 years of age, and her biopsy showed that this was “venous”, and not a cancer. In her mid 30s, poor vision led to the diagnosis of a retinal abnormality. At the age of 50 years, she had nephrectomy for renal cancer, and cysts were seen in the remaining kidney. The husband and father of patients 1 and 2, respectively, suffered poor vision from his early 30s, and was diagnosed with retinal angiomas and detachment. He died of a heart attack at 41 years of age. The deceased brother of patient 3 presented with paraparesis due to renal cancer metastasis at 38 years of age; no retinal disorder was diagnosed. The father of patient 1 had occipital surgery for a brain tumour, which was not a cancer, but “involved blood vessels.” He became blind due to retinal disease. He died of a heart attack at 70 years of age. The deceased great aunt of patient 2, an obligate heterozygote from the pedigree, was not known to have had VHL-related disease. According to incomplete anecdotal information, it is likely that the great aunt was nonpenetrant at the level of overt symptomatic disease.

**Figure 2:**
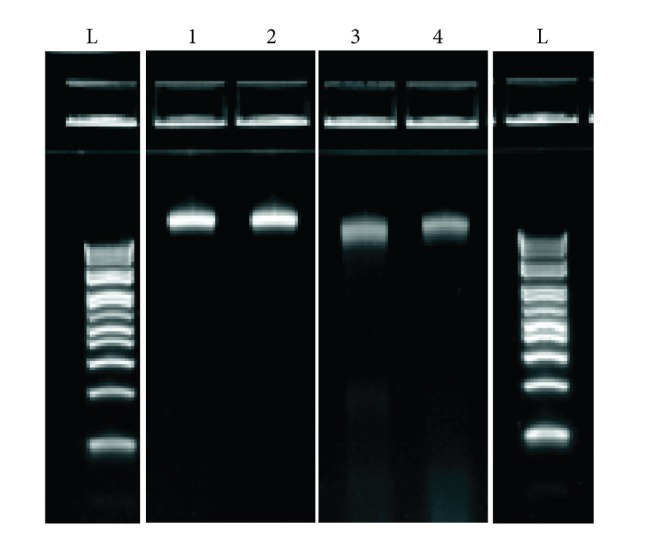
Gel electrophoresis of genomic DNAs isolated from the patients in this study. One to four refer to DNAs extracted from the blood samples of patients 1 and 2 and the saliva samples of patients 3 and 4, respectively. L refers to a 1 kb plus ladder.

In order to identify the “family-specific *VHL* gene mutation,” an affected family member (shown as patient 3 in Figure 1, and Table 1) in Iraq offered to provide a sample for testing. Another apparently unaffected family member (identified as patient 4 in Figure 1) also requested testing, should a mutation be identified. These patients provided saliva samples, while patients 1 and 2 (see Figure 1) were based in New Zealand and they provided peripheral blood samples. The DNAs isolated from the saliva samples had 260 nm/280 nm ratios of 1.78–1.85 and 260 nm/230 nm ratios of 0.98–1.2; the latter ratio is considered to reflect salt and alcohol contamination and has been reported by others for whole saliva samples [10]. Electrophoretic separation of the DNAs isolated from the saliva and blood samples showed the DNAs to be high in molecular weight, but with some degradation of those DNAs isolated from saliva (Figure 2).

### 3.2. Molecular Analysis

**Figure 3:**
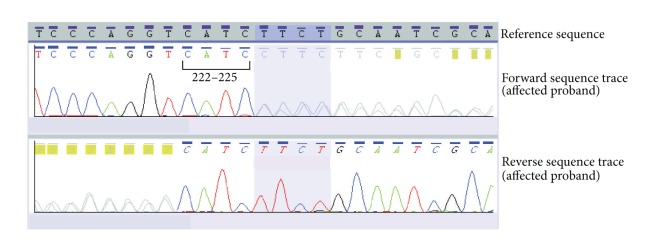
Sequence electropherograms showing the c.222_225dupCATC *VHL* gene mutation identified in the proband.

**Figure 4:**
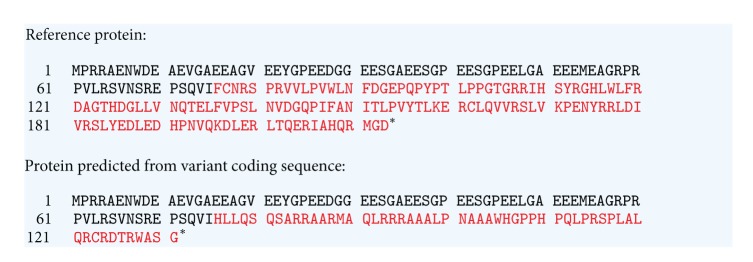
Predicted amino acid sequences of the proteins expressed from the wild type and mutant *VHL* gene transcripts (NM_000551.3). The wild type (reference) protein and the expected variant protein expressed from the *VHL* gene transcript (Refseq accession number: NM_000551.3) are shown. The single letter amino acid code has been used; the amino acids indicated in red identify those downstream of amino acid 75, and the asterisk indicates translation termination. This figure has been adapted from the output that was achieved by accessing the Mutalyzer 2.0.beta27 website (https://mutalyzer.nl/check) and inserting the transcript accession number and the DNA alteration, c.222_225dup.

**Figure 5:**
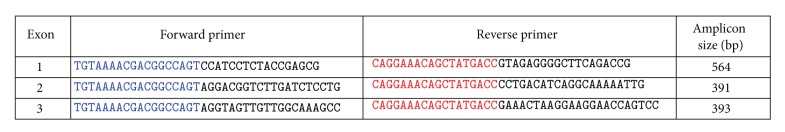
Primers for the amplication of all coding exons of the *VHL* gene (Refseq accession number NM_000551.3). Blue and red coloured bases represent M13 sequences that tail the forward and reverse primers, respectively.

Conventional PCR and bidirectional sequence analysis of all coding exons of the *VHL* gene in the affected individual (patient 3) identified a heterozygous variant c.222_225dupCATC (p.Phe76HisfsX57) in exon 1 (see Figures 3 and 4).

DNA extracted from the peripheral blood of patients 1 and 2 and from the saliva of patient 4 was analysed for the presence of this variant. The variant was proved to be absent in all of these patients.

## 4. Discussion

The use of saliva as the DNA sample source allowed for the simple collection and transportation of the samples from Iraq to New Zealand. The saliva samples were stable through a range of temperatures during the course of postage from Iraq to New Zealand and allowed the isolation of DNA that was of sufficient quality and quantity for our analysis of the *VHL* gene. 

We were dependant upon the accuracy of the anecdotal clinical information of the affected family member in Iraq as supplied to us, but the description was consistent with VHL as the correct diagnosis. The New Zealand family's informant, individual 4 in Figure 1, was the brother of the proband and a physician.

A duplication variant was detected in exon 1 of the *VHL* gene in the proband, c.222_225dupCATC (p.Phe76HisfsX57). This variant is not listed in the online Human Gene Mutation Database Pro (HGMD Pro, https://portal.biobase-international.com/hgmd/pro/start.php) or the VHL Universal Mutation Database (UMD, http://www.umd.be/VHL/) and does not appear to be reported in the literature. However, it leads to the premature truncation of the pVHL30 protein and so is of a type which is typically pathogenic. 

pVHL30 comprises a seven-stranded *β*-sandwich, termed the *β* domain (amino acids 71–78, 84–89, 95–97, 105–112, 116–121, 129-130, and 147–15), together with a three helix cluster termed the *α* domain (amino acids 158–169, 183–189 and 194–206); see http://www.rcsb.org/pdb/explore/explore.do?structureId=1LM8) The *β* domain provides a binding site for hydroxyproline peptide-containing ligands. The highly conserved amino acids Trp88, Trp89, and Trp 117 of pVHL30 are involved in the van der Waals contacts with the pyrrolidone ring of hydroxyprolines such as those found in HIF-1*α* and the large subunit of RNA polymerase II. The *α* domain binds Elongin C, and in association with other proteins, forms a ubiquitin-protein ligase [11–15]. The effect of the frameshift mutation reported here is to remove critical tryptophan amino acids in the *β* domain and also the entire carboxyl terminal *α* domain. A mutation of this type is expected to be associated with a type 1 VHL syndrome, which carries a low risk of pheochromocytoma [8, 16]. Truncating mutations have also been shown to lead to a higher risk of RCC in VHL patients, and the proband had been diagnosed with kidney cancer [9]. 

As international mobility of families becomes common, and often between countries of greatly differing standards of medical facilities and of access to molecular laboratory testing, clinical genetic services are increasingly dependent upon data and DNA coming from abroad. The case we present here demonstrates the ease of genetic testing over international borders using saliva kits and also identifies a novel *VHL* gene variant.

## Figures and Tables

**Table 1:** Patient information.

Patient	Age at testing (years)	Gender	Country	Symptoms
1	53	Female	New Zealand	None
2	27	Male	New Zealand	None; deceased father had a possible diagnosis of VHL
3	51	Female	Iraq	Kidney cancer, brain (?cerebellar haemangioblastoma) cancer, eye (?retinal angioma) disease
4	49	Male	Iraq	None
